# Preselection of robust radiomic features does not improve outcome modelling in non-small cell lung cancer based on clinical routine FDG-PET imaging

**DOI:** 10.1186/s13550-021-00809-3

**Published:** 2021-08-21

**Authors:** Carol Oliveira, Florian Amstutz, Diem Vuong, Marta Bogowicz, Martin Hüllner, Robert Foerster, Lucas Basler, Christina Schröder, Eric I. Eboulet, Miklos Pless, Sandra Thierstein, Solange Peters, Sven Hillinger, Stephanie Tanadini-Lang, Matthias Guckenberger

**Affiliations:** 1 Department of Radiation Oncology, University Hospital Zurich, University of Zurich, Zurich, Switzerland; 2 Division of Radiation Oncology, Cancer Center of Southeastern Ontario, Queen’s University, Kingston, ON Canada; 3 Department of Nuclear Medicine, University Hospital Zurich, University of Zurich, Zurich, Switzerland; 4 Swiss Group for Clinical Cancer Research (SAKK) Coordinating Center, Bern, Switzerland; 5 Department of Medical Oncology, Kantonsspital Winterthur, Winterthur, Switzerland; 6 Department of Oncology, Centre Hospitalier Universitaire Vaudois (CHUV), Lausanne, Switzerland; 7 Department of Thoracic Surgery, University Hospital Zurich, University of Zurich, Zurich, Switzerland

**Keywords:** Multi-centre, Radiomics, Lung cancer, PET, Robust

## Abstract

**Background:**

Radiomics is a promising tool for identifying imaging-based biomarkers. Radiomics-based models are often trained on single-institution datasets; however, multi-centre imaging datasets are preferred for external generalizability owing to the influence of inter-institutional scanning differences and acquisition settings. The study aim was to determine the value of preselection of robust radiomic features in routine clinical positron emission tomography (PET) images to predict clinical outcomes in locally advanced non-small cell lung cancer (NSCLC).

**Methods:**

A total of 1404 primary tumour radiomic features were extracted from pre-treatment [^18^F]fluorodeoxyglucose (FDG)-PET scans of stage IIIA/N2 or IIIB NSCLC patients using a training cohort (*n* = 79; prospective Swiss multi-centre randomized phase III trial SAKK 16/00; 16 centres) and an internal validation cohort (*n* = 31; single centre). Robustness studies investigating delineation variation, attenuation correction and motion were performed (intraclass correlation coefficient threshold > 0.9). Two 12-/24-month event-free survival (EFS) and overall survival (OS) logistic regression models were trained using standardized imaging: (1) with robust features alone and (2) with all available features. Models were then validated using fivefold cross-validation, and validation on a separate single-centre dataset. Model performance was assessed using area under the receiver operating characteristic curve (AUC).

**Results:**

Robustness studies identified 179 stable features (13%), with 25% stable features for 3D versus 4D acquisition, 31% for attenuation correction and 78% for delineation. Univariable analysis found no significant robust features predicting 12-/24-month EFS and 12-month OS (*p* value > 0.076). Prognostic models without robust preselection performed well for 12-month EFS in training (AUC = 0.73) and validation (AUC = 0.74). Patient stratification into two risk groups based on 12-month EFS was significant for training (*p* value = 0.02) and validation cohorts (*p* value = 0.03).

**Conclusions:**

A PET-based radiomics model using a standardized, multi-centre dataset to predict EFS in locally advanced NSCLC was successfully established and validated with good performance. Prediction models with robust feature preselection were unsuccessful, indicating the need for a standardized imaging protocol.

**Supplementary Information:**

The online version contains supplementary material available at 10.1186/s13550-021-00809-3.

## Background

Imaging is a fundamental tool in medicine and especially in personalized medicine [1]. Medical imaging in oncology is important for diagnosis, staging, treatment and response assessment. However, data extracted from radiological imaging have traditionally largely been qualitative, limiting the role of imaging in precision medicine. Only recently, imaging has been recognized as non-invasive biomarkers by extracting a large amount of data using mathematical image-analysis methodologies [2, 3]. Imaging-based biomarkers have therefore found their way into prognostic models to predict clinical outcome in investigative settings [4].

Radiomics refers to the extraction of a large number of quantitative features from medical images [5]. In addition to using standard imaging tumour characteristics, such as tumour volume, contrast enhancement, or maximum standardized uptake value (SUV_max_), numerous other parameters, which may not be visible to the naked eye, may be extracted with radiomics [6, 7]. Radiomic features quantitatively describe different tissue characteristics, such as grey-value distribution or inter-pixel relationships. They can be categorized into shape, intensity, texture and filter-based (wavelet) features [5]. Radiomics has been applied to a variety of imaging, including computed tomography (CT), magnetic resonance imaging (MRI) and positron emission tomography (PET), with good prognostic power for different entities in a research setting [8–13].

Biomarkers are objective, quantifiable characteristics of a biological process [14]. Biomarkers are commonly seen as molecular markers, measured in biological samples, such as blood or tissue. However, medical parameters and indexes, such as heart rate or blood oxygen saturation, and imaging-based biomarkers can function as biomarkers following the same principles. Radiomics, as image-based biomarkers, has emerged as a novel approach in precision medicine, as it allows for thorough multi-modality image assessment accounting for intratumoural heterogeneity and change over time in a non-invasive, fast and affordable way by extracting a large number of phenotypic tumour characteristics from routine imaging [3, 15–18]. Radiomic features are currently being used in research studies, but before becoming part of clinical decision making, further validation and qualification are needed [19]. Addressing variability of PET imaging and radiomics methodology has been called for by several studies [20, 21].

One of the major strengths of radiomic biomarkers is that they can be extracted non-invasively, from routinely acquired imaging, which makes it cost-effective [5]. However, the protocols in these routinely acquired images have been mostly optimized for qualitative assessment. Therefore, image quality often varies among centres or among scanners, as well as over time. In PET imaging, several studies showed high instability rates of radiomic features depending for example on tumour motion, delineation, image reconstruction or image resampling [4, 22–27]. These robustness studies were based on phantom investigations or repeated imaging of the same patients. They are however often limited to investigation of one single cause of feature instability. Moreover, the robustness studies mostly focused on the stability of the features without its implication on the prognostic power of PET radiomics.

This study aims to investigate the value of preselection of robust radiomic features in clinical routine PET images, which are subject to the aforementioned variations, to predict clinical outcomes in locally advanced non-small cell lung cancer (NSCLC). If indeed prognostic models based on robust PET-based radiomic features alone are feasible, image acquisition standardization may not be necessary, allowing for wider implementation of the methodology and higher generalizability of the results. On the other hand, it might be that robust preselection removes features with high prognostic values and image standardization is therefore the preferred option. Patient-based datasets were used to evaluate three sources for radiomic feature instability: tumour delineation, attenuation correction and tumour motion. Consecutively, throughout stable features for tumour delineation, attenuation correction and tumour motion were tested for prediction of event-free survival (EFS) and overall survival (OS) using standardized image datasets. For comparability, two sets of models using standardized datasets were built, with the first set using all available features, independent of their robustness, and the second one using robust features only, to test the hypothesis whether EFS and OS prediction using robust features in locally advanced NSCLC using a real-life highly heterogenous dataset is feasible, or if image standardization is required.

## Methods

### Workflow

**Fig. 1:**
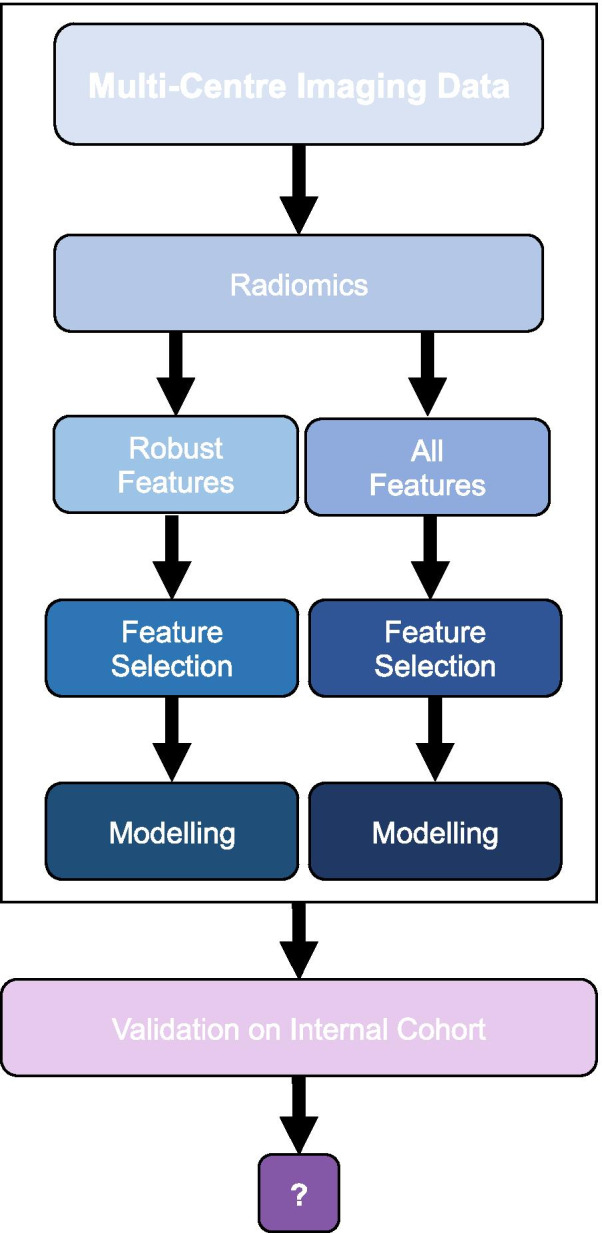
Workflow. Robust radiomic features (delineation, attenuation correction, 3D vs. 4D acquisition) were identified. Two prognostic models, using robust features only and using all available features, respectively, were constructed for 12- and 24-month event-free survival (EFS) and overall survival (OS) based on a standardized multi-centre imaging dataset. Model performance was validated using a separate internal validation cohort

**Fig. 2:**
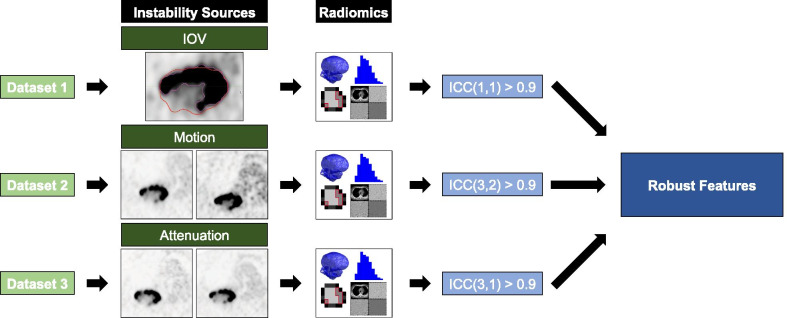
Robustness studies. The influence of delineation variability or image was investigated using either a pair of scans for each patient as it is the case for the PET/CT/PET/MR and motion comparison, or different methods of defining the regions of interest (ROI), as applied for the delineation study. Radiomics calculation was performed on each individual ROI. Robust features were determined using the intraclass correlation coefficient (ICC)

Robust radiomic features (delineation, attenuation correction, motion) were identified in different subsets of clinical [^18^F]fluorodeoxyglucose (FDG)-PET images (Figs. 1 and 2). Two predictive models, first using robust features only and second using all available features, were used for prediction of EFS and OS at 12, 18 and 24 months. For redundancy reasons, results were reported for 12- and 24-month outcomes only (18-month results are listed in Additional file 1: Supplement A1). Models were validated using an independent single-centre validation cohort. Finally, performance of all models was assessed.

### Prognostic modelling

#### Studied cohorts

The training cohort (TC) was derived from a prospective Swiss multi-centre randomized phase III trial (SAKK 16/00) on IIIA/N2 NSCLC patients [28]. In this multi-modality treatment comparison trial, patients underwent neoadjuvant chemotherapy or chemoradiotherapy prior to surgery (43 vs. 36). Radiomic features of pre-treatment PET scans of primary tumours of ≥ 72 voxels (after resizing to 5.5 mm cubic voxels) were included in the TC (*n* = 79). Small tumours (< 72 voxels) were excluded to ensure meaningful wavelet feature calculations. In the validation cohort (VC), separate pre-treatment PET scans of 31 stage IIIA/N2 or stage IIIB NSCLC patients were included. Patients were treated with induction chemotherapy or chemoradiotherapy in curative intent (30 vs. 1) prior to surgery at the University Hospital Zurich (USZ). Initial datasets consisted of 103 and 38 patients, of which 24 and 7 cases were excluded due to small tumour size in the TC and VC, respectively. Median EFS was 13.3 and 16.3 months, and median OS was 55.6 and 53.6 months, for the TC and VC, respectively.

Ethics amendment approvals were received from all involved Swiss canton ethics committees and informed consent was obtained from all individual participants. Ethics amendments requesting inclusion of the current study were documented in Additional file 1: Supplement A2. Ethics board approval and written consent were obtained for the VC as well (KEK ZH 2018-02405).

Patients were staged according to the 6th edition of the TNM classification as defined in the SAKK 16/00 trial. The two outcomes of interest were EFS and OS. For the TC, clinical outcomes were defined according to the SAKK 16/00 trial, with EFS being time from randomization to relapse, progression, second tumour, or death, whichever occurred first, and OS being defined by death [28]. The same definitions were applied for the VC with the date of diagnosis being used instead of the date of randomization.

#### Imaging, delineation and radiomics

The analysis was performed based on pre-treatment [^18^F]FDG-PET/CT imaging. The TC imaging dataset consisted of imaging from different centres (*n* = 16). The VC dataset included 31 pre-treatment PET scans. The imaging datasets were standardized for tumour delineation, attenuation correction and tumour motion. Additional file 1: Supplement A3 lists technical details on the used PET scanners.

Primary tumours were contoured based on CT and PET images manually by a medical student and radiation oncology trainee using MIM VISTA (Version 6.7.9., MIM Software Inc., Cleveland, USA). The contours were checked for consistency by a senior radiation oncologist. Initial registration of the PET and CT scans was optimized manually. Contouring was based on the PET signal and CT findings. Images were resized to cubic voxels (5.5 mm) with linear interpolation. A fixed bin size of 0.25 SUV was used for texture calculation. A Hounsfield unit (HU) range of − 300 to 200 was used to exclude bone and lung tissue from the analysis based on CT intensities and PET/CT registration.

### Robustness studies

Three potential sources of radiomic feature instability (tumour motion, attenuation correction and tumour delineation) were studied individually while two factors were kept constant in each dataset used for robustness studies. Each dataset consisted of 9–10 separate, single-centre IIIA/N2 or IIIB NSCLC patients. First, three delineation methods were investigated: manual delineation and semi-automated delineation (threshold based and gradient based). Contours were created in the MIM VISTA software (Version 6.7.9., MIM Software Inc., Cleveland, USA). Primary lesions were manually delineated using fused PET/CT images. For semi-automated segmentation, threshold and gradient tools in MIM VISTA were used. The threshold was adapted for individual patients and ranged from 27 to 41% of SUV_max_ [29]. Second, motion effects were evaluated using free-breathing 3D data acquisition and gated 4D phase acquisition. In the gated acquisition, the quiescent phase of the respiratory cycle was chosen for comparison as it was considered the most stable phase [4]. Third, attenuation correction was evaluated by comparing PET radiomic features from PET/CT and PET/MR scans of the same patient according to Vuong et al. [29]. The summary of imaging, reconstruction and delineation protocols for the three datasets is presented in Table 1.

**Table 1:** Overview of image acquisition characteristics

Robustness study	Delineation (3 methods)	Attenuation correction		Motion	
		CT based	MR based	Average	Gating
Number of patients	9	9		10	
Scanner manufacturer	GE Healthcare, Waukesha	GE Healthcare, Waukesha		GE Healthcare, Waukesha	GE Healthcare, Waukesha
Scanner model	Discovery 690	Discovery 690	SIGNA PET/MR	SIGNA PET/MR	SIGNA PET/MR
Reconstruction method	VPFXS	VPFXS	VPFXS	VPFXS	VPFXS
Attenuation correction	MR based: LAVA-flex pulse sequence	CT based	MR based: LAVA-Flex pulse sequence	MR based: LAVA-Flex pulse sequence	
Time delay between FDG injection and PET scan start (min)	71.5–92.5	71.5–92.5	40.3–117.6	57.1–75.9	36.8–82.0
Injected activity (MBq)	181.2–252.3	181.2–252.3		136.2–259.3	
Acquisition type	3D	3D		3D	4D (phase-gated)
Time per bed position (min)	2	2	2	2	2
Resolution (mm)	2.73 × 2.73 × 3.27	2.73 × 2.73 × 3.27	2.34 × 2.34 × 2.78	2.34 × 2.34 × 2.78	2.34 × 2.34 × 2.78
Delineation	Gradient-based threshold-based manual	Gradient based	Gradient based	Gradient based	Gradient based
Intraclass correlation coefficient (ICC)	ICC(1,1)	ICC(3,1)		ICC(3,2)	

PET image dataset acquisition characteristics including reconstruction and delineation protocols for the three datasets are listed

### Radiomics calculation

Radiomics calculation was performed with an in-house developed radiomics software *Z*-rad implemented in Python programming language (Version 2.7.10). Resampling of the images to 5.5 mm cubic voxels was performed using linear interpolation. A fixed bin size of 0.25 standardized uptake value (SUV) was chosen. In total, 1404 radiomic features were calculated, i.e. shape (*n* = 18), intensity (*n* = 17), texture (*n* = 137) and wavelets (*n* = 1232) (for further details see https://medical-physics-usz.github.io/). Shape, intensity and texture feature definition were standardized to the image biomarker standardization initiative (IBSI) [18]. To compare the radiomic features within a certain robustness dataset, the intraclass correlation coefficient (ICC) was calculated [30]. Type of ICC used for each individual robustness study is listed in Table 1. An ICC larger than 0.9 was considered stable.

### Statistical analysis

PET radiomics prognostic models were trained to predict EFS and OS at 12, 18 and 24 months as defined by the SAKK 16/00 trial protocol [28]. Models were trained separately using all 1404 features and robust features alone, referred to as standard and robust models, respectively. Principal component analysis (PCA) was performed to group correlated features. The Horn method [31] was used to select the number of retained components. Features were grouped based on their correlations to the principal component group. As a group surrogate, the feature with the largest area under receiver operating characteristics curve (AUC) in the univariable analysis was selected. Only features with a *p* value < 0.05 were considered. Final feature selection was performed in the multivariable logistic regression with backward selection of variables based on Akaike information criterion (AIC). Performance of the models was tested in fivefold cross-validation using the TC.

Per clinical endpoint (EFS, OS) and feature set (standard, robust), models performing best in the TC, defined as best trade-off between the largest average AUC and the smallest range of AUCs in the cross-validation folds, were validated in the independent VC. To further investigate effects of different robustness factors on the prognostic value PET radiomics, a set of features with AUC > 0.6 in both training and validation cohorts was selected (12-month EFS and 12-month OS). Within this set, the percentage of features robust against each of the 3 factors (tumour motion, attenuation and delineation) was reported separately. Model building, validation and comparison were performed using R (Version 3.5.3), with packages base, survival [32], survcomp [33], boot [34], pROC [35] and glmnet [36].

## Results

### Robustness studies

**Fig. 3:**
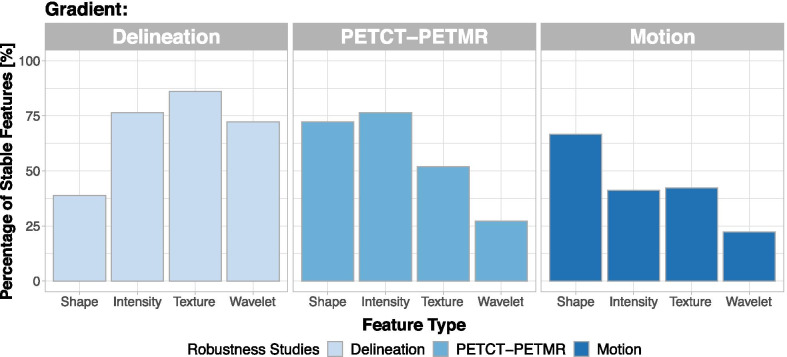
Robust radiomic features. Percentage of stable features for each of the robustness studies, i.e. the interobserver delineation variability, PET/CT/PET/MR, and respiratory motion are depicted. For each robustness study, the percentage of stable features is shown for the four feature types: shape, intensity, texture and wavelet. The feature type with the highest stability differed between the studies, with the lowest stability in the delineation study being shape, and wavelet in the PET/CT / PET/MR and motion studies

Robustness results are shown in Fig. 3. The majority of features (78%) were stable with regard to delineation differences. Attenuation correction method and motion had a stronger influence on feature stability. Thirty-one per cent of the features were not affected by attenuation correction, and 25% were stable regardless of motion. Altogether, only 13% of the features were robust in respect to all three studied factors. Shape features showed poor reproducibility in the delineation dataset, but they were robust against motion and attenuation correction. On the other hand, wavelet features were highly dependent on motion and attenuation correction, but they showed high stability in the delineation dataset. The overlap of the three studies is visualized with Venn diagrams (Additional file 1: Supplement A4). Overall, the overlap of robust features was small among all feature types, with shape and intensity features showing the highest overlap (> 35% of robust features stable), followed by texture (23.4%) and wavelet features (10.3%).

### Prognostic modelling

#### Robust features

Univariable analysis identified no significant robust features for EFS at 12 and 24 months, and OS at 24 months. Only one significant robust feature was identified for OS at 12 months: HHL NGLDM dependence count entropy, a wavelet feature (Table 2). This 12-month OS model using robust features only was tested in the validation cohort, but did not perform well (AUC = 0.53, 95% confidence interval (95% CI) 0.26–0.81).

**Table 2:** Results of the multivariable analysis

Outcome	Radiomic features (feature type)	Robustness delineation (ICC)	Robustness attenuation correction (ICC)	Robustness motion (ICC)	AUC training (range)	AUC validation (95% CI)
12-month EFS	LHL coefficient of variation (wavelet)	0.79	0.81	0.00	0.73 (0.65–0.81)	0.74 (0.55–0.93)
	LHH neighbouring grey-level dependence matrix high dependence high grey-level emphasis (wavelet)	0.91	0.35	0.92		
	HLH skewness (wavelet)	0.58	0.37	0.43		
24-month EFS	HLH mean (wavelet)	0.49	0.17	0	0.74 (0.58–0.94)	
	LHH neighbouring grey-level dependence matrix high dependence high grey-level emphasis (wavelet)	0.91	0.35	0.92		
12-month OS	LLH skewness (wavelet)	0.74	0.06	0.47	0.85 (0.6–1)	0.67 (0.43–0.91)
	HLL kurtosis (wavelet)	0.93	0.93	0.75		
	HHL skewness (wavelet)	0.85	0.00	0.49		
	LLH grey-level run length matrix short run high grey-level emphasis (wavelet)	1.00	0.83	0.93		
24-month OS	HLL skewness (wavelet)	0.82	0.91	0.39	0.69 (0.57–0.8)	
12-month OS robust preselection	HHL NGLDM dependence count entropy (wavelet)	0.95	0.98	0.96	0.67 (0.46–0.85)	0.53 (0.26–0.81)

Results multivariable analysis for EFS and OS. Radiomic features were selected using backward selection. Good classification performances of models without robust preselection were observed for the training cohort (AUC = 0.69–0.85) and the validation cohort (AUC = 0.67–0.74). Performance of the robust model was moderate in training (AUC = 0.67) and weak in validation (AUC = 0.53)

The different impact of the robustness factors on the prognostic value of PET radiomics was observed. For 12-month EFS, 9 features showed AUC > 0.6 in both training and validation, but only 22% were stable against motion and delineation, and 0% were stable against attenuation. Similarly, for 12-month OS, 116 prognostic features were identified, from which 68% were stable against delineation and 19% were stable against attenuation and motion.

#### All features

Prognostic models using features irrespective of their robustness were identified for EFS and OS at all timepoints (Table 2). The final multivariable models consisted of 3 and 2 radiomic features for 12- and 24-month EFS, respectively. For 12- and 24-month OS, 4 and 1 significant radiomic features were identified, respectively.

**Fig. 4:**
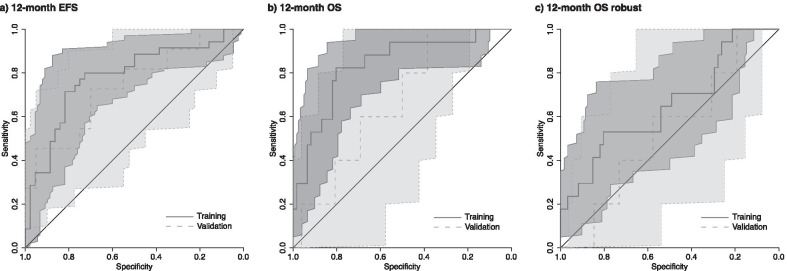
ROC curves. Receiver operating characteristic (ROC) curves with 95%-CI for **a** 12-month EFS, **b** 12-month OS and **c** robust 12-month OS for training (dark grey, solid lines) and validation cohorts (light grey, interrupted lines) are shown. Only the 12-month EFS model trained on the entire feature set without robust preselection was successful in the validation cohort

The best trade-off between the largest average AUC and smallest AUC range in cross-validation was observed for 12-month EFS (average AUC = 0.73) and 12-month OS models (average AUC = 0.85). Only the 12-month EFS model was successfully validated, resulting in AUC = 0.74 with a 95% CI of 0.55–0.93 (Table 2 and Fig. 4).

**Fig. 5:**
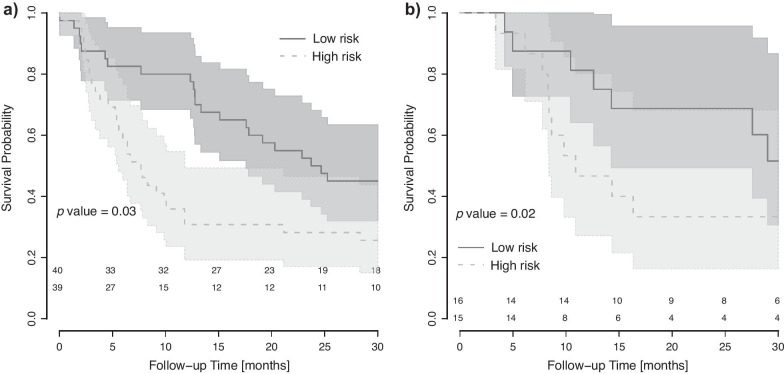
Patient stratification based on 12-month EFS. Patient stratification into high risk (light grey, interrupted lines, 95%-CI in light grey) and low risk (dark grey, solid lines, 95%-CI in dark grey) was significant for 12-month EFS: **a** training cohort, *p* value = 0.03 and **b** validation cohort, *p* value = 0.02

The probabilities from the 12-month EFS model were used to create Kaplan–Meier curves, based on the median in the TC (Fig. 5). The split was significant, both in the TC (g-rho test*, p* value = 0.03) and VC (*p* value = 0.02). The low-risk group had significantly longer median EFS, 8 versus 25 months in the TC and 11 versus 29 months in the VC.

## Discussion

Event-free survival prediction of locally advanced NSCLC based on radiomics models was successfully performed using multi-centre clinical routine FDG-PET datasets and validated using an independent internal VC. The 12-month EFS model using LHL coefficient of variation (wavelet feature), LHH neighbouring grey-level dependence matrix high dependence high grey-level emphasis (wavelet feature) and HLH skewness (wavelet feature) resulted in the highest AUC in validation (AUC = 0.74). Increased LHL coefficient of variation as well as decreased LHH neighbouring grey-level dependence matrix high dependence high grey-level emphasis and HLH skewness were associated with no events at 12 months, suggesting that more heterogeneous FDG uptake pattern is associated with worse prognosis, which has been observed in other studies [37, 38]. Except for 12-month OS, models using preselected robust features only, could not be built using our feature selection scheme. The 12-month OS model with robust feature preselection yielded poor performance (AUC = 0.53). This indicates that robust feature preselection excluded a prohibitive large number of important radiomic features from the prognostic models. It can therefore be extrapolated that a clean, standardized dataset with similar imaging quality, especially in terms of attenuation correction and motion compensation, is required for generalizable and transferable EFS and OS prediction allowing for all available features to be potentially included.

While a majority of radiomics studies in locally advanced NSCLC assessed the prognostic value of CT-based features, our study is adding to the emerging body of the literature on PET-based models [4, 16, 22, 23, 37–44]. Although it is challenging to systematically compare NSCLC PET-based radiomics studies at this time due to different cohorts in terms of stage and treatment, as well a different radiomic, biological and clinical features tested, varied statistical methodology employed and common scarcity of model validation, some studies indicate a link between higher heterogeneity and worse prognosis [37, 38]. Interpretation of radiomic features as biomarkers may be challenging for clinicians, given their large number and not yet well-understood association with tumour characteristics and biological processes. Imaging variations not only affect the robustness of features but may also influence the association between imaging features and the underlying tumour activity distribution. In a phantom study, the association between PET radiomic features in the lung and underlying intratumoural heterogeneity was shown to be strongly influenced by image acquisition and PET imaging reconstruction [45]. While these results need additional validation for wavelet features, they point to an inherent problem in radiomics. In addition to modelling strategies, where our results indicate improved modelling performance with standardized imaging, feature association with tumour activity distribution can be maintained across all patients when using standardized imaging settings. On the other hand, simpler imaging-based biomarkers have already found their way into clinical practice, such as the SUV_max_ [4]. However, conventional PET-parameters, such as SUV_max_, SUV_peak_ or SUV_mean_ were not selected into our final models, as they showed lower prognostic value than more complex radiomic features. While this is in agreement with previous publications, other studies have shown some prognostic value of SUV descriptors [37, 38, 42, 43, 46]. In our study, a comprehensive number of radiomic features (*n* = 1404) were tested for their predictive value, including shape, texture, intensity and wavelet features. This is in contrast to other studies, where a more selective number of features was assessed [16, 38–40, 42–44]. This comprehensive approach allowed to identify the prognostic value of wavelet features, which is different from other studies, where mostly textural features were included in predictive models. The majority of PET-based outcome prediction studies in NSCLC used single-institution imaging, thereby minimizing heterogeneity within datasets. Ohri et al. tested 43 textural features, metabolic tumour volume (MTV) and SUV_max_, using a multi-centre trial dataset, and identified one texture feature, SumMean, an indicator of homogeneity, as prognostic for OS among patients with large primary tumours [40]. The authors hypothesize that the strong association may indicate feature robustness in their multi-institutional dataset, thereby increasing generalizability [40]. A second multi-centre study by Arshad et al. claimed that slice thickness and matrix size did not significantly affect the predictive feature vector discovered (FVX), concluding that robustness of FVX permits this variable to be applied in multi-institutional studies [42]. In our study, standardized multi-centre PET imaging was used to allow for generalizable comparison of prediction models, using robust feature preselection and all available features.

After the initial success of radiomic models for prediction of different outcomes, robustness of the features started to be frequently discussed in the context of multi-centre validation [3, 4]. By its design, PET imaging characterizes tumour biology by the use of different radiopharmaceuticals, which makes it a perfect modality for prognosis assessment. However, an inherent complex nature of image acquisition (processes linked to radiotracer uptake, signal acquisition, image reconstruction and postprocessing) makes it a challenging modality to analyse with quantitative methods. Several initiatives exist worldwide to improve the comparability between images acquired in different institutions [47–51]. Rapid development of detector technology and reconstruction methods makes collection of large and homogenous datasets difficult. Recently, in the context of quantitative texture analysis, specialized PET radiomics phantoms have been investigated to depict heterogeneity of PET tracer uptake [4]. While phantoms may facilitate the analysis of a larger number of confounding factors in a single study, to date, most radiomics robustness studies have focused on a single factor only.

The secondary investigation focus of our study was the robustness of radiomic features in presence of multiple confounding factors (delineation variability, attenuation correction and tumour motion). Only 13% of the features were robust against all three studied factors. In the individual studies, a higher percentage of stable features was observed for delineation variability (78%) than for attenuation correction (31%) and motion difference (25%). This translated into a larger number of stable and prognostic features (AUC > 0.6) in the presence of interobserver delineation variability than different attenuation correction and motion compensation. However, the types of stable features differed considerably between robustness studies. For delineation variability, shape features were found to be the least stable ones, which is expected since shape is directly affected by different delineations. On the other hand, wavelet and texture features, which are less dependent on boundary definition, displayed a higher number of robust features. In the case of attenuation correction and motion studies, findings were the opposite. Shape features were less affected by these factors, but the number of stable texture and wavelet features was lower. Texture and wavelet features constitute a majority of the studied features, and thus, the overall percentage of features stable against attenuation correction and motion was low. Impact of delineation variability and motion on robustness of PET radiomic features was also studied by other groups and results were comparable to ours. For delineation variability, a study by Leijenar et al. reported robustness of 91% of the features for NSCLC patients [23]. The value is 13% higher than in our study; however, they used a less strict criterium of ICC > 0.8 [23]. A study conducted by Takeda et al. found 86% (ICC > 0.8) robust features for interobserver variability, but only seven radiomic features were investigated [39]. The impact of motion on feature robustness was studied by Oliver et al. finding that the percentage of stable features between respiration-gated images and averaged images over all phases was 26.2% [22]. This value is very close to the one obtained in our study; however, a detailed comparison is not possible as stability was not defined using ICC.

Strengths of our study include a prospective multi-centre training dataset, a large number of tested radiomic features, radiomic feature robustness assessment, model validation on a separate dataset and stratification by disease stage and treatment. This study adds to our recent publication on CT-based radiomics to predict OS of locally advanced NSCLC and shows that in contrast to CT-based radiomics, prognostic PET-based radiomics models require harmonized PET imaging, as robust feature preselection excluded a prohibitive large number of important radiomic features from the prognostic models [52]. Generalizability of our models is therefore restricted to patients who underwent similar PET imaging. The importance of using a clean imaging dataset was further illustrated by a recent phantom study by Ger et al. which found that most radiomic feature values showed good reliability when PET imaging protocol parameters were within clinically used ranges, but that interscanner variability was similar to interpatient variability, leading the authors to caution radiomics analyses on patients scanned on different PET scanners [53]. While our results support the need for harmonized PET imaging and we advocate for standardization of protocols, the impact assessment of different PET scanners was not the thrust of our study. Often heterogeneity by different PET scanners is unavoidable in a clinical setting. Another limitation of our study may be the restricted reproducibility of our results, as they depend on an in-house software. However, our software was benchmarked within the Image Biomarker Standardization Initiative (IBSI) [18]. While the TC consisted of prospectively acquired data following a strict trial protocol, the VC consisted of a retrospective dataset potentially allowing for introduction of patient selection bias. Further, while our study stratified for stage and surgical treatment, it did not include other clinical or molecular outcome predictors, such a smoking habits or epidermal growth factor receptor (EGFR) status, which may influence EFS and OS and could hypothetically improve the models’ predictive power. However, inclusion of clinical parameters was out of scope of this study, as we aimed to investigate the optimal modelling strategy for robust multi-centre PET radiomics models. Similarly, only the primary tumour was taken into account in our study, as it is commonly the case in radiomics studies and in accordance with the study objectives. While we were able to categorize the patient cohort into low-risk and high-risk groups, biological correlation of these groups and individual radiomic factors used in the prognostic models remain unknown. In addition, sample size is another limitation of our study, which is related to availability of data. However, methodological steps were taken to address potential related statistical issues such as using PCA to reduce dimensionality, excluding correlated features and evaluating model performance in cross-validation using the TC as well as testing the models in a completely separate VC as recommended by the Transparent Reporting of a Multivariable Prediction Model for Individual Prognosis or Diagnosis (TRIPOD) statement [54]. Additional data is needed to further validate the study findings. Further, although this study investigated more than one robustness factor, the set of factors was still limited. However, two factors (motion and attenuation correction) are linked to new developments in the field, motion correction (with the recent advent of data-driven deviceless gating techniques) and PET/MR hybrid devices, and thus make them relevant factors to be studied. A recent study showed that randomization of voxel intensities had an impact on model prognostication [55]. Since the goal of this study was to simulate a real-world environment, this aspect remained outside the scope of this work. Another limitation is the fact that all the robustness studies were conducted on different datasets with a small number of patients. This may be solved in the future with introduction of highly specialized heterogenous PET phantoms. Only one setting of radiomics calculation parameters was investigated (bin size, voxel size, HU range). The results of robustness studies might be influenced by this choice but considering the low number of patients in the robustness studies and moderate size of datasets in the prognostic modelling step, it was deemed important to limit the number of extracted features.

In conclusion, a PET-based radiomics model using multi-centre datasets to predict EFS in locally advanced NSCLC was successfully established. However, PET acquisition standardization is necessary, as prediction models using robust features alone could not be built or showed poor performance. Therefore, a standardized dataset with similar image acquisition and reconstruction is required for EFS prediction based on PET-based radiomics models.

## Supplementary Information

**Additional file 1.Supplement A1:** 18-Month Results. **Supplement A2:** Training Cohort Ethics Board Amendment Documents. **Supplement A3:** PET-Specific Information from the Training Cohort and Validation Cohort. **Supplement A4:** Venn Diagrams of the Robustness Studies.

## Data Availability

The datasets used and/or analysed during the current study are available from the corresponding author on reasonable request (data sharing agreements needed). In-house developed radiomics software Z-rad implemented in Python programming language (Version 2.7.10) was used. For further details, see https://medical-physics-usz.github.io/*.* Software is available upon request to the authors.

## Abbreviations

AIC: Akaike information criterion
AUC: Area under the receiver operating characteristic curve
CT: Computed tomography
EFS: Event-free survival
EGFR: Epidermal growth factor receptor
FDG: Fluorodeoxyglucose
FVX: Predictive feature vector discovered
HU: Hounsfield unit
IBSI: Image biomarker standardization initiative
ICC: Intraclass correlation coefficient
MRI: Magnetic resonance imaging
MTV: Metabolic tumour volume
NSCLC: Non-small cell lung cancer
OS: Overall survival
PCA: Principal component analysis
PET: Positron emission tomography
ROC: Receiver operating characteristic
ROI: Regions of interest
SUV: Standardized uptake value
TC: Training cohort
VC: Validation cohort
