# Supplementary material for: Preselection of robust radiomic features does not improve outcome modelling in non-small cell lung cancer based on clinical routine FDG-PET imaging
Source: EJNMMI Res. 2021 Aug 21;11:79. doi: 10.1186/s13550-021-00809-3 (PMC8380219; doi:10.1186/s13550-021-00809-3)

## Additional file

Supplement A1: 18-Month Results

Univariable analysis found no significant robust features predicting 18-month EFS and OS (*p*-value >0.05). Predictive models using all available features found below results for prediction of EFS and OS at 18 months:

| **Outcome** | **Radiomic Features (feature type)** | **AUC Training**  **(range)** |
| --- | --- | --- |
| 18-month EFS | - LLL grey level size zone matrix small zone low grey level emphasis (wavelet) - HLH mean (wavelet) - LLH grey level size zone matrix small zone low grey level emphasis (wavelet) | 0.71 (0.56-0.91) |
| 18-month OS | - HHL skewness (wavelet) - LLH skewness (wavelet) | 0.73 (0.55-0.86) |

Supplement A2: Training Cohort Ethics Board Amendment Documents

- EKNZ PB_2016-01071
- KEK ZH PB_2016-00412
- KEK Bern PB_2016-01072
- CER-VD PB_2016-01078
- CCER PB_2016-01073
- EKOS PB_2016-01075
- Comitato Etico Cantonale Bellinzona PB_2016-01077

Supplement A3: PET-Specific Information from the Training Cohort and Validation Cohort

| **Cohort** | **Mean dose [MBq] (range)** | **Mean injection-scan delay [min] (range)** | **In-plane resolution (range)** | **Scanner manufacturer**  **[count]** | **Slice thickness [mm] (range)** |
| --- | --- | --- | --- | --- | --- |
| Training cohort  (n = 79) | 346.8  (211.5 -  699.0) | 68.41  (36.5 - 205.38) | 4.43  (2.73 - 5.47) | GE Medical Systems: 55  Phillips Medical Systems: 3  Siemens: 14  Unknown: 7 | 3.38  (2 - 5) |
| Validation cohort  (n = 31) | 321.7  (239.4 – 404.3) | 65.26  (42.05 - 110.03) | 4.69  (2.73 - 5.47) | GE Medical Systems: 24  Siemens: 7 | 2  (2 - 3.27) |

Supplement A4: Venn Diagrams of the Robustness Studies
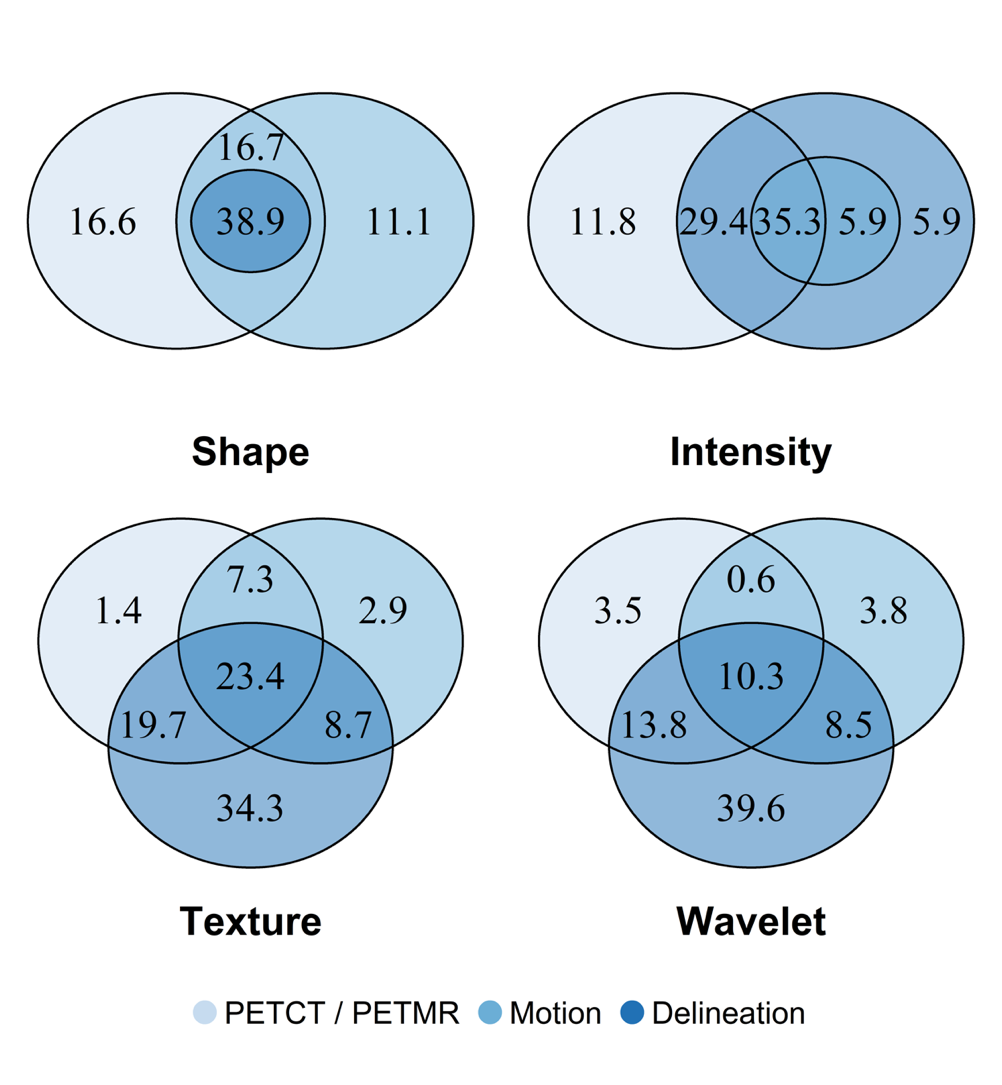

Supplement: Supplementary file 1 — Additional file 1.Supplement A1: 18-Month Results. Supplement A2: Training Cohort Ethics Board Amendment Documents. Supplement A3: PET-Specific Information from the Training Cohort and Validation Cohort. Supplement A4: Venn Diagrams of the Robustness Studies. [file 13550_2021_809_MOESM1_ESM.docx]
